# What Is My Cancer Risk? How Internet-Based Cancer Risk Assessment Tools Communicate Individualized Risk Estimates to the Public: Content Analysis

**DOI:** 10.2196/jmir.1222

**Published:** 2009-07-31

**Authors:** Erika A Waters, Helen W Sullivan, Wendy Nelson, Bradford W Hesse

**Affiliations:** ^2^Basic and Biobehavioral Research BranchNational Cancer Institute; ^1^Health Communication and Informatics Research BranchNational Cancer Institute

**Keywords:** Risk, risk assessment, communication, risk communication, perception, risk perception, calculators, programmable, risk calculator, Internet, online, cancer

## Abstract

**Background:**

Internet-based cancer risk assessment tools have the potential to inform the public about cancer risk and promote risk-reducing behaviors. However, poorly communicated information on these websites may result in unintended adverse health outcomes.

**Objective:**

This study examined whether: (1) Internet-based cancer risk assessment tools use risk communication formats that facilitate comprehension and reduce bias (as identified by the empirical literature); (2) the use of these formats varies by website affiliation; and (3) the websites provided information necessary to evaluate the quality of the risk estimate.

**Methods:**

A content analysis of Internet-based cancer risk assessment tools was conducted. The terms *calculate cancer risk, cancer risk calculator, estimate cancer risk, assess cancer risk,* and *cancer risk assessment* were searched using three search engines. We identified 47 risk assessment tools and coded each according to standardized criteria. We calculated simple frequencies on all coding categories and performed crosstabulations but did not conduct formal statistical analysis due to small cell sizes.

**Results:**

Use of risk communication formats that facilitate comprehension and reduce bias varied widely (eg, 30% of websites [14/47] provided absolute and comparative risk information but 83% [39/47] provided safety messages). Use of formats that facilitate comprehension varied by website affiliation and communication strategy (eg, only 8.3% [1/12] websites affiliated with the health care industry provided absolute and comparative risk information, but 83% [5/6] of websites affiliated with a governmental organization did so). Only 53% (25/47) of websites provided information about the statistical model or the peer-reviewed literature that was used to calculate the risk estimate.

**Conclusion:**

Internet-based cancer risk assessment tools varied in their use of risk communication formats that facilitate comprehension and reduce bias. Formats that are difficult to understand may cause people to misperceive their cancer risk and consequently take inappropriate action.

## Introduction

Many laypeople obtain individualized cancer risk estimates from Internet-based risk assessment tools. Data from the 2005 Health Information National Trends Survey (HINTS), which comprises a representative sample of the United States’ adult population, reveal that approximately 25% of people have used the Internet to seek information about cancer [1]. Although the proportion of people who sought information about their cancer risk is not known, use of online cancer risk assessment tools is common. For example, the *Your Disease Risk* website averaged nearly 2000 visitors per day in 2006 (personal communication by Graham Colditz, 2006), and the National Cancer Institute’s Breast Cancer Risk Assessment Tool averaged over 1200 hits per day in 2007 (personal communication by Rick Manrow, 2008). The purpose of providing people with individualized risk estimates is to encourage them to engage in health-promoting behaviors [2,3], such as using the health care system appropriately, making good medical decisions, engaging in health screening, avoiding tobacco use, engaging in physical activity, and eating a healthy diet. To achieve this, it is crucial that the information be presented in a way that facilitates comprehension and does not bias risk perceptions [4].

Gurmankin Levy and colleagues [5] examined the quality of risk estimates for 13 breast cancer risk assessment websites. Many of these risk assessment tools used different risk factors to calculate risk, and in some cases excluded well-established risk factors, such as age at first live birth. Consequently, it is not surprising that the risk estimates provided for a particular risk profile varied across assessment tools. These authors [5] also found that many Internet-based risk assessment tools did not provide sufficient information to evaluate website quality. For example, some websites did not contain information about institutional affiliations or identify the statistical model used to calculate risk [5,6]. Because millions of people seek cancer risk information from Internet sources [1], the public health implications of inaccurate or inadequate risk communication are clear. Risk assessment tools that provide incorrect information may lead people to experience negative outcomes, such as seeking too much or too little medical care.

Even when risk assessment tools provide accurate risk estimates, the information needs to be presented clearly. People do not always understand probabilistic information [7-11], even though they often report wanting to use it to make medical decisions [12]. The dilemma is exacerbated by the fact that risk perceptions, risk comprehension, and decision making can be affected by the ways in which probability information is presented [4,13-18]. For example, arrays of stick figures (also referred to as “pictographs” and “icon arrays”) can make it easier for people to understand the effects of hypothetical medical treatments and can reduce the undue influence that side effects have on hypothetical treatment decisions [19]. Misperceiving one’s risk of illness may result in poor health decisions. For example, a woman who overestimates her risk of developing cervical cancer might undergo excessive screening, but she might also be so overwhelmed with anxiety that she avoids screening entirely.

A number of researchers have tested various ways to communicate probabilistic risk information [9,14,16,20-22]. According to these studies, the optimal risk communication format depends on what the communication is expected to accomplish. For example, presenting a drug’s benefit only as a relative risk reduction (“reduce the overall mortality by 20.3%”) can be more effective in persuading physicians to prescribe a drug than presenting only absolute risk reduction information (“reduce overall mortality from 7.8%...to...6.3%”) [23] (page 123). However, if the goal is education rather than persuasion, providing both absolute and relative information is most effective [24]. Thus, no single risk communication format will be appropriate in all situations and under all conditions.

If the goal of Internet-based risk assessment tools is to assist people in making good decisions about their health, they must communicate probabilistic risk information in a way that facilitates comprehension and reduces biased interpretations [4]. A large and growing literature on risk communication permits us to infer which formats are likely to be most useful in helping people understand cancer risk estimates (Table 1). However, it is unknown how these formats interact with each other to influence perceptions of risk. Consequently, Table 1 should not be viewed as a checklist of required criteria, but instead as a list of important factors to take into consideration when developing a tool. Just as readers would not use a statistical test without understanding its underlying principles, they should not develop a risk assessment tool without having some rudimentary understanding of how different risk communication formats might affect risk perceptions and comprehension. Comprehensive reviews of the complex issues surrounding probabilistic risk communication can be found at [21,22,24-27].

**Table 1:** Communication formats that reduce bias and facilitate comprehension of probabilistic risk estimates^a^

Risk Communication Format and Selected Relevant Citations	Why the Recommended Format Is Important When the Communication Goal Is to Educate and Inform
Describe the risk using words and numbers [20,22].	Using words only is ambiguous because people assign different numeric values to the same label (eg, “small” can mean “2%” to some people and “10%” to others). Using numbers only is problematic due to the population’s low levels of numeracy (ie, the ability to use numeric information) and a lack of contextual information (eg, Should a 7% lifetime risk of breast cancer be considered a high risk or a low risk?).
Communicate numeric risk as N in 1000 or as a percentage [16,17,20,22].	Risk comprehension is highest when risk estimates are presented as a percentage or as N in 1000, compared to other formats like the number-needed-to-treat or odds ratios. However, both recommended formats have drawbacks. The N in 1000 format can encourage people to overemphasize risk by “imagining the numerator,” but the percentage format is more difficult to use when conducting complex calculations (eg, the probability of a woman having breast cancer given a positive mammogram).
Provide absolute and comparative risk information [20,25,28-30], but see [24].	Providing both absolute and comparative information helps people determine the amount of importance that they should place on the risk and guides them in making informed decisions about their behavior. For example, telling a woman that she has a 5% 5-year risk of developing breast cancer might not be meaningful unless she recognizes that this means that she is at above average risk. However, telling people only that they are at below-average risk might reduce motivation to engage in preventive behavior.
Compare cancer risk to the risk of other hazards [22].	Helping people understand where their risk of cancer falls in relation to other hazards such as heart disease, being struck by lightening, and being in a car accident allows them to place the risk in context and thereby help them determine where to invest their limited time, energy, and economic resources.
Frame the risk in positive and negative terms [18,20,25].	Framing the risk in negative terms only (eg, “Your risk of cancer is 5%”) places focus only on the negative outcome and might result in exaggerated risk perceptions. Adding positive framing (eg, “This means you have a 95% chance of not getting cancer) helps participants place the risk in context.
Specify the duration of risk [20,25].	Specifying whether the risk estimate is applicable to the next 5 years, 10 years, or over the visitor’s lifetime is essential to help them place the risk in context and determine how much they should be concerned about the event. For example, a 7% risk of breast cancer would be more worrisome if it was applicable to the next 5 years than over one’s lifetime.
Provide safety messages and risk reduction strategies [31-33].	Informing people how to reduce their risk is an essential component of risk communication messages, particularly for individuals who have not learned risk reduction strategies previously. Providing risk information without such safety messages may undermine risk communication efforts by encouraging people to control their fear (eg, by trying to ignore the risk) rather than encouraging people to control the danger (eg, by engaging in appropriate health behaviors).
Include a visual display of risk [20,22,26].	Using a visual display can increase comprehension of risk information. However, care must be taken to avoid biasing perceptions of risk (eg, displays that focus attention on the number of people affected by a disease can exaggerate a risk compared to displays that include information about the number of people affected and the number of people who are not affected).
Acknowledge that the risk estimate contains an element of uncertainty [22].	Individualized risk estimates are based on statistical modeling of population-level data. Consequently, they always contain a level of uncertainty. Informing the audience of this fact is essential to prevent them from attributing an unreasonable degree of certainty to the estimate.

^a^These formats can be implemented with varying levels of success and might not be equally effective in all situations. Additional examples of each format are located in Table 5.

 This study describes the risk communication formats that Internet-based cancer risk assessment tools use to convey individualized risk information to the public. This study asks two questions that, to our knowledge, have not been addressed previously: (1) Do the tools use risk communication formats that have been empirically shown to facilitate comprehension, and (2) Does the use of these formats vary by website affiliation? Following Gurmankin Levy and colleagues [5], we also examined whether the websites provided the basic information necessary to evaluate the quality of the risk estimate (ie, the statistical model or peer-reviewed citations).

## Methods

### Overview

During October 2007 we conducted an Internet search to identify websites that provided individualized cancer risk assessment. The search was conducted by entering the search terms *calculate cancer risk, cancer risk calculator, estimate cancer risk, assess cancer risk,* and *cancer risk assessment* into the Google, MSN, and Yahoo! search engines. These search engines accounted for 82.6% of all Internet searches that originated in the United States in 2006 [34]. To locate the Internet-based cancer risk assessment tools, a total of 1500 websites were examined (ie, the first 100 search results for each of the five search terms, for each of the three search engines).

Out of the 1500 websites examined, we identified 51 websites that gave specific cancer risk estimates. Forty-four of the identified websites were unique interactive websites that provided individualized cancer risk estimates. These websites required visitors to enter information about their status on several cancer risk factors. Seven of the identified websites were non-interactive; they stratified risk information by two or three variables (eg, lung cancer risk by smoking status and gender). These non-interactive websites were included because they provided more specific risk information than general population data. Of these 51 websites, four were excluded because they required information seekers to download a software program (n = 2), provide a mailing address (n = 1), or provide payment (n = 1) before obtaining results. A total of 47 websites were evaluated (Table 2).

**Table 2:** Websites hosting cancer risk assessment tools (WebCite^®^ links are listed below the original URL)

1.	Breast Link http://www.breastlink.com/default.aspx Archived by WebCite® at http://www.webcitation.org/5gBFlHdu9
2.	CancerRiskInfo.com http://www.cancerriskinfo.com/ Archived by WebCite® at http://www.webcitation.org/5g6prLoiq
3.	Carefirst Blue Cross Blue Shield http://carefirst.staywellsolutionsonline.com/RelatedItems/42,BreastCancerRisk Archived by WebCite® at http://www.webcitation.org/5gBHb6xdn
4.	Center for Cancer Quality Assurance and Professional Education http://qap.sdsu.edu/screening/breastcancer/bda/flowcharts/risk_algo1.html Archival by WebCite® prohibited by website.
5.	Claxton Hepburn Medical Center http://www.chmed.org/breastca.htm Archived by WebCite® at http://www.webcitation.org/5gBHInllZ
6.	Cornell University http://envirocancer.cornell.edu/factsheet/diet/fs49.BCRisk.cfm Archived by WebCite® at http://www.webcitation.org/5gBGdq722
7.	Dermatology Imaging Center http://www.dermatologyimaging.com/skincancertest.html Archived by WebCite® at http://www.webcitation.org/5gBGrY1lI
8.	Divine http://www.divine.ca/en/breast-cancer-corner/breast-cancer-risk-calculator/c_244/ Archived by WebCite® at http://www.webcitation.org/5gBHOBQOw
9.	Dr. Halls MD http://www.halls.md/breast/risk.htm Archived by WebCite® at http://www.webcitation.org/5g6orRIOY
10.	EBSCO Publishing http://calculators.epnet.com/?docid=healthcalculators/breastcancer/precalcdoc&token=b0c3eb60-99e5-4038-bc04-819fded5c1d6&DeliveryContext=healthlibrary&CollectionIID=446&frame=&rooturl= Archived by WebCite® at http://www.webcitation.org/5gBIDVLXM
11.	Estronaut.com http://www.estronaut.com/a/breastInteractive2.htm Archived by WebCite® at http://www.webcitation.org/5g6pLGN9G
12.	Fairview Health Services http://www.fairview.org/staywell/assess_load.aspx?ContentTypeId=42&ContentId=OvarianCancerRisk Archived by WebCite® at http://www.webcitation.org/5gBHTLDA0
13.	Fred Hutchinson Cancer Research Center http://www.compass.fhcrc.org/edrnnci/bin/calculator/main.asp?t=prostate&sub=disclaimer&v=prostate&m=&x=Prostate%20Cancer Clicking this link now redirects visitors to the University of Texas Science Center in San Antonio, Texas: http://deb.uthscsa.edu/URORiskCalc/Pages/uroriskcalc.jsp Archived by WebCite® at http://www.webcitation.org/5gBGIO1yJ
14.	Hotflash! Menopause Matters http://www.families-first.com/hotflash/news/mayoquiz6.htm Archived by WebCite® at http://www.webcitation.org/5g6s0slTC
15.	Imaginis http://imaginis.com/breasthealth/bc_risks2.asp Archived by WebCite® at http://www.webcitation.org/5gBGVk6Tl
16.	iVillage.com http://cancer.health.ivillage.com/tools/assessment_index.cfm Archived by WebCite® at http://www.webcitation.org/5g6pJnqwO
17.	Little Company of Mary Hospital and Health Care Centers http://pursuingpainfreecancer.com/breast_test.php Archived by WebCite® at http://www.webcitation.org/5gBHvAIVP
18.	McGill University http://www.mcgill.ca/cancerepi/society/calculate/ Archived by WebCite® at http://www.webcitation.org/5g6p4wfuN
19.	MD Anderson Cancer Center http://www2.mdanderson.org/app/risk/ Archived by WebCite® at http://www.webcitation.org/5gBH8xzEu
20.	Merck and Co. https://www.merck.healthinkonline.com/merckTools/AssessMerckSourceBreastCancer.asp Archived by WebCite® at http://www.webcitation.org/5gBFra58Q
21.	Memorial Sloan Kettering Cancer Center http://www.mskcc.org/mskcc/html/12463.cfm Archived by WebCite® at http://www.webcitation.org/5g6oz4wBH
22.	Men’s Health Forum http://www.malehealth.co.uk/userpage1.cfm?item_id=117&pop=326 Archived by WebCite® at http://www.webcitation.org/5g6q64w6Y
23.	Mesoblog http://www.mesoblog.org/risk-calculator.php Archived by WebCite® at http://www.webcitation.org/5gBHQNRgC
24.	National Breast and Ovarian Cancer Centre http://www.nbcc.org.au/risk/yourrisk.html Archived by WebCite® at http://www.webcitation.org/5gBHLKXwC
25.	National Surgical Adjuvant Breast and Bowel Project http://www.breastcancerprevention.org/raf_source.asp Archived by WebCite® at http://www.webcitation.org/5g6p0aimY
26.	Northeast Health Systems http://www.nhshealth.org/index.cfm?Action=Education.BreastCancerQuiz Original link no longer valid. Non-interactive version of the site can be found at: http://web.archive.org/web/20070826153816/http://www.nhshealth.org/index.cfm?Action=Education.BreastCancerQuiz
27.	Norton Healthcare http://norton.convergencehealth.com/DesktopDefault.aspx?tabid=820&id=653 Archival by WebCite® prohibited by website.
28.	Ohio State University Medical Center http://www.jamesline.com/patientsandvisitors/prevention/cancergenetics/?ref=medicalnews Archived by WebCite® at http://www.webcitation.org/5gBHhJ5LK
29.	Penn State Hershey Cancer Institute http://www.hmc.psu.edu/cancer/outreach_education/community/cancer_risk_assessments/cancer_risk_assessment.htm Link no longer valid; WebCite® citation unavailable.
30.	Prostate Cancer Research Foundation of Canada http://www.prostatecancer.ca/english/prostate_owners_manual/risk_factors/risk/ Archived by WebCite® at http://www.webcitation.org/5g6rslb1L
31.	Radon Seal http://www.radonseal.com/radon-health-risks.htm Archived by WebCite® at http://www.webcitation.org/5gBFg7aJS
32.	Real Age http://www.realage.com/health_guides/BreastCancer/introduction.asp Archived by WebCite® at http://www.webcitation.org/5gBHjAMpu
33.	Shannon Health http://shannon.convergencehealth.com/DesktopDefault.aspx?tabid=1390&id=644 Archival by WebCite® prohibited by website.
34.	Siteman Cancer Center (not the Your Disease Risk website) http://www.siteman.wustl.edu/crc.aspx?id=459 Archived by WebCite® at http://www.webcitation.org/5gBGnKmVx
35.	St. John’s Hospital https://www.healthawareservices.com/nahrs/index.htm?hospID=19&moduleName=lungAware Archived by WebCite® at http://www.webcitation.org/5gBHqIAQU
36.	Susan Love http://www.susanlovemd.com/breastcancer/content.asp?L2=2&L3=2&SID=140 Archived by WebCite® at http://www.webcitation.org/5gBGc9yJy
37.	Urology Channel http://www.urologychannel.com/HealthProfiler/healthpro_psaageRace.shtml Archived by WebCite® at http://www.webcitation.org/5g6rqRUJo
38.	US Environmental Protection Agency http://www.epa.gov/radon/risk_assessment.html Archived by WebCite® at http://www.webcitation.org/5g6s2DYZK
39.	US National Cancer Institute (Breast Cancer) http://www.cancer.gov/bcrisktool/ Archived by WebCite® at http://www.webcitation.org/5g6ouFL9F
40.	US National Cancer Institute (Melanoma) http://www.cancer.gov/melanomarisktool/ Archived by WebCite® at http://www.webcitation.org/5g6owaI5h
41.	US National Cancer Institute (Thyroid Cancer) http://ntsi131.nci.nih.gov/ Archived by WebCite® at http://www.webcitation.org/5g6p2gkq6
42.	US National Cancer Institute and the Centers for Disease Control http://www.smokefree.gov/smokersrisk/index.asp Archived by WebCite® at http://www.webcitation.org/5g6pI9GAQ
43.	Vizilite http://www.vizilite.com/patient_site/risk_assessment/ Original link no longer valid. Non-interactive version of the site can be found at: http://web.archive.org/web/20080105051853/http://www.vizilite.com/patient_site/risk_assessment/
44.	Women’s Cancer Network http://www.wcn.org/interior.cfm?diseaseid=13&featureid=3 Original link no longer valid. Non-interactive version of the site can be found at: http://web.archive.org/web/20071228025221/http://www.wcn.org/interior.cfm?diseaseid=13&featureid=3
45.	World Information Service on Energy http://www.wise-uranium.org/rdcum.html Archived by WebCite® at http://www.webcitation.org/5g6q2Y2Gf
46.	Wyoming Valley Healthcare System http://www.wvhc.staywellsolutionsonline.com/InteractiveTools/RiskAssessments/42,BreastCancerRisk Archived by WebCite® at http://www.webcitation.org/5g6s7Apal
47.	Your Disease Risk http://www.yourdiseaserisk.org/ Archived by WebCite® at http://www.webcitation.org/5epfwmhdn

Standardized coding criteria were developed (Table 3) and each website was coded independently by two of the authors (EW and HS). The first 15 websites were used to calibrate the coding procedure, and inconsistencies in the remaining 32 websites were resolved through discussion. Inter-rater reliability was high (κ = 86.4). If information about the statistical model used to calculate the risk could not be found on the website, EW attempted to obtain it by contacting the website’s developer. Two attempts to reach the developers were made over a four-week period.

**Table 3:** Website coding criteria

Coding Category			Example
**General website characteristics**			
	Organ site		Breast
	Type of affiliation		Educational institution
	**Accessibility to lay audiences**		
		Intended audience	Lay people
		Contains undefined terminology	Biopsy
		Non-English version	Spanish
**Risk communication strategies**			
	Words		“Your risk is low.”
	Numbers		“Your risk is 2%.”
	Format of numeric information		Percent, frequency (n in 1000), frequency (1 in N)
	Absolute risk		“Your risk is low.” OR “Your risk is 2%.”
	Comparative risk (other people)		“Your risk is higher than average.”
	Comparative risk (other hazards)		“Your risk of getting cancer is 12%. The risk of being injured in a car accident is 10%.”
	Positive framing		“Your risk is 2 in 100. Your chances of not getting cancer are 98 in 100.”
	Duration of risk		“Your 5-year risk is...”
	Safety message/Risk reduction strategy		“Stop smoking.”
	Visual display		Bar graph, Line graph, Table
	Acknowledges uncertainty		“Just because you’re at high risk doesn’t mean you’ll definitely get cancer.” OR “This estimate is based on information obtained from the population and your actual risk might be different.”
**Quality evaluation elements**			
	Information about the statistical model		“This website uses the Gail Model.”
	Peer-reviewed citation		“Harvard Report on Cancer Prevention, Volume IV: Harvard Cancer Risk Index, Cancer Causes and Control, Volume 11:477-488, 2000.”


### Analyses

General website characteristics, risk communication formats, and the presence of information about the quality of the risk estimate were examined by calculating simple frequencies on all coding categories. The number and percentage of websites that used formats that had the most empirical support for facilitating comprehension and reducing bias was recorded (see Table 1 for a list of formats). Affiliation-based differences in the use of formats were examined using crosstabulations. Formal statistical analysis and significance testing was not possible due to small cell sizes.

## Results

### General Website Characteristics

The general characteristics of the websites are described in Table 4. There are two areas of particular interest. First, the three most common organ sites for which websites provided assessments (breast, lung, and colorectal cancer) coincide with the three leading causes of cancer mortality in the United States [35]. The second item of interest is the widespread use of technical language. Although laypeople were the intended audience for nearly all of the sites, an overwhelming majority did not define medical and technical terms such as “biopsy,” “DCIS,” “mastectomy,” or “radon progeny” (Table 4).

**Table 4:** General website characteristics (N = 47)

Website Characteristic		Example	n	%
**Organ site^a, b^**				
	Bladder		4	8.5
	Breast		27	57.5
	Cancer (general)		5	10.6
	Cervical		8	17.0
	Colorectal		10	21.3
	Gastrointestinal		5	10.6
	Kidney		5	10.6
	Lung		12	25.5
	Ovarian		9	19.2
	Pancreatic		3	6.4
	Prostate		9	19.2
	Skin/Melanoma		6	12.8
	Other	Thyroid	8	17.0
**Website affiliation**				
	Government	National Cancer Institute	6	12.8
	Educational institution^c^	McGill University	3	6.4
	Cancer center^c^	Memorial Sloan-Kettering	8	17.0
	Health care industry	CareFirst Blue Cross Blue Shield	12	25.5
	Advocacy/non-profit	Women’s Cancer Network	6	12.8
	Health portal^d^	RealAge.com; Imaginis.com	5	10.6
	Commercial industry	RadonSeal	3	6.4
	Other/unspecified	Dr. Halls; EBSCO publishing	4	8.5
**Accessibility to lay audiences^b^**				
	Intended audience: Lay people		42	89.4
	Contains undefined terminology	Biopsy	39	83.0
	Non-English version	Spanish	3	6.4

^a^Websites varied in the number of organ sites for which they provided risk assessments. Most provided assessments for only one cancer site, but others provided assessments for more than one organ site (between 1 and 14 additional organ sites, depending on the website).

^b^The total N in *organ site, quality evaluation elements,* and *accessibility to lay audiences* categories does not sum to 47 because the individual elements within each category were not mutually exclusive.

^c^Cancer centers are often located within educational institutions, but the objectives and methods of these two types of institutions might differ. For this reason, assessment tools that were developed by cancer centers that were affiliated with educational institutions were coded as cancer centers.

^d^Health portals are websites that contain information about a variety of medical conditions and/or health issues. WebMD.com [36] is an example of a health portal, although it did not host a cancer risk assessment tool at the time of the study.

### Formats for Communicating Individualized Cancer Risk Estimates

In general, few websites used risk communication formats that facilitate comprehension of probabilistic information (Table 5). Few websites provided the risk estimate as numbers and words, or described how the information seeker’s cancer risk compared to the risk of experiencing other hazards. Only three websites framed the risk positively (eg, “998 chances in 1000 that you will not develop cancer”), and slightly less than half informed participants of the duration of the risk estimate (eg, 5-year risk). Approximately one-third of the websites provided a visual display. However, some risk communication formats were used widely. Seventeen of the 21 websites that provided any numeric information did so using the percentage format, six used the “N in 1000” format, and three used both. Twenty-four websites compared the information seeker’s cancer risk to other people’s risk, and fourteen of these also provided the absolute risk. An overwhelming majority of the websites provided information seekers with safety messages like “stop smoking.” Slightly more than half of the websites made at least one statement acknowledging that the estimate contained some degree of uncertainty.

**Table 5:** Risk communication formats used by Internet-based cancer risk assessment tools to communicate individualized risk estimates (N = 47)

Risk Communication Format		Example	n	%
**Words or numbers**				
	Words only	Your risk is low.	24	51.1
	Numbers only	Your risk is 2%.	16	34.0
	*Both*^a^	*Your risk is 2%. This is a low risk.*	*5*	*10.6*
	Neither	You may only need to continue screening.	2	4.3
**Type of numeric information^b, c^**				
	*Percent*	*Your risk is 2%.*	*17*	*81.0*
	*Frequency (n in 1000)*	*Your risk is 20 in 1000.*	*6*	*28.6*
	Frequency (1 in N)	Your risk is 10 in 500.	4	19.1
	Relative risk ratio	Your risk is 2 times higher than average.	2	9.5
	Odds	Your odds of getting cancer are 2:98.	1	4.8
**Risk estimate as absolute or comparative information**				
	Absolute risk only	Your risk is low.” OR “Your risk is 2%.	21	44.7
	Comparative risk only	Your risk is higher than average.	10	21.3
	*Absolute and comparative risk*	*Your risk is 2%. This is below average. OR Your risk is 2%. The average risk is 3%.*	*14*	*29.8*
	Neither absolute nor comparative risk	You may only need to continue screening.	2	4.3
**Types of comparative risk information**				
	*Compared to other people only (not hazards)*	*Your risk is higher than average.*	*21*	*44.7*
	*Compared to other people and hazards*	*Your risk of getting cancer is 12%, which is higher than average. The risk of being injured in a car accident is 10%.*	*3*	*6.4*
	No comparison information		23	48.9
**Contextual information^b^**				
	*Positive framing*	*Your risk is 2 in 100. This means your chances of not getting cancer are 98 in 100.*	*3*	*6.4*
	*Duration of risk*	*Your 5-year risk is...*	*23*	*48.9*
**Safety messages**				
	*At least one*	*Stop smoking*	*39*	*83.0*
	None		8	17.0
**Visual display**				
	*At least one*	*Bar graph, line graph, table*	*18*	*38.3*
	None		29	61.7
**Acknowledgment of uncertainty: Estimate is...^b^**				
	only an estimate	Your actual risk might be different.	14	29.8
	probabilistic	High risk doesn’t mean you’ll get cancer.	15	31.9
	based on population	This estimate is based on data from large clinical trials.	8	17.0
	*Any acknowledgment*		*25*	*53.2*

^a^In general, the formats printed in italics are associated with increased comprehension and reduced bias of risk information. For comprehensive reviews see [21,22,24-27].

^b^The individual elements within the categories *type of numeric information, additional information, and acknowledgment of uncertainty* were not mutually exclusive.

^c^This category is restricted to the 21 websites that provided numeric risk information.

The use of risk communication formats that facilitate comprehension and reduce bias varied by website affiliation (Table 6). For example, websites affiliated with the health care industry were the least likely to communicate risk as percentages or as frequencies (1 of 12 websites) and to provide both absolute and comparative risk information (1 of 12 websites). Websites affiliated with cancer centers were the least likely to provide information about the duration of the risk estimate (1 of 8 websites) and to make at least one statement acknowledging that the estimate contained uncertainty (1 of 8 websites). Health portals were the least likely to provide a safety message or risk reduction strategies (2 of 5 websites). As mentioned in the Analyses section, formal statistical analyses and significance testing could not be performed due to small cell sizes (ie, out of 72 possible cells, only eight contained five or more websites and fifteen included no websites; see Table 6).

**Table 6:** Website affiliation-based variations in the use of risk communication formats that facilitate comprehension and reduce bias (N = 47)

Supported Format	---------------------------- Affiliation ----------------------------							
	Government (N = 6) n (%)	Educational Institution (N = 3) n (%)	Cancer Center (N = 8) n (%)	Health Care Industry (N = 12) n (%)	Advocacy/ Non-profit (N = 6) n (%)	Health Portal (N = 5) n (%)	Commercial (N = 3) n (%)	Other (N = 4) n (%)
Risk estimate as numbers and words^a^	1 (16.7)	0 (0.0)	0 (0.0)	1 (8.3)	0 (0.0)	1 (20.0)	0 (0.0)	2 (50.0)
Risk estimate as percent or N in 1000^b^	5 (83.3)	2 (66.7)	2 (25.0)	1 (8.3)	2 (33.3)	4 (80.0)	1 (33.3)	3 (75.5)
Absolute and comparative risk information	5 (83.3)	2 (66.7)	1 (12.5)	1 (8.3)	1 (16.7)	2 (40.0)	1 (33.3)	1 (25.0)
Risk compared to other hazards	0 (0.0)	1 (33.3)	0 (0.0)	0 (0.0)	0 (0.0)	1 (20.0)	1 (33.3)	0 (0.0)
Positive framing	2 (33.3)	0 (0.0)	0 (0.0)	0 (0.0)	0 (0.0)	1 (20.0)	0 (0.0)	0 (0.0)
Duration of risk	6 (100.0)	2 (66.7)	1 (12.5)	4 (33.3)	2 (33.3)	4 (80.0)	1 (33.3)	3 (75.0)
Safety messages	5 (83.3)	2 (66.7)	7 (87.5)	12 (100.0)	4 (66.7)	2 (40.0)	3 (100.0)	4 (100.0)
Visual display	3 (50.0)	2 (66.7)	2 (25.0)	3 (25.0)	2 (33.3)	3 (60.0)	2 (66.7)	1 (25.0)
Any acknowledgment of uncertainty	6 (100.0)	2 (66.7)	2 (25.0)	6 (50.0)	2 (33.3)	3 (60.0)	1 (33.3)	3 (75.0)

^a^Percentages are the percent of websites within a given affiliation that contain a particular element (eg, 1 of 6 websites affiliated with government agencies provided risk estimates as numbers and words).

^b^Includes websites that provided risk as numbers only and as numbers and words.

### Information Necessary to Evaluate Website Quality

The number of websites that provided information to help visitors evaluate the quality of the risk estimate was limited. Only 25 of the 47 (53.2%) websites provided either information about the statistical model used to calculate the risk or peer-reviewed citations, where such information could be found. Only 13 websites (27.7%) provided both a description of the model and peer-reviewed citations. One of the authors (EW) attempted to contact all 22 of the websites that did not provide any information about the model. Of the 15 websites that provided a valid email address or phone number, only 8 responded to either the first or second inquiry, and only 2 were able to provide the information.

## Discussion

Internet-based cancer risk assessment tools can provide cancer risk and prevention information to millions of people worldwide. Effective risk communication has the potential to reduce cancer morbidity and mortality by motivating people to engage in healthy behaviors. However, poorly communicated risk information could mislead people or frighten them unduly, resulting in maladaptive health behaviors [37], such as over- or under-utilization of health care services.

In October 2007, 47 Internet-based cancer risk assessment tools provided individualized cancer risk information to the public. Almost half (20 out of 47) of these websites were developed by cancer centers (n = 8) and the health care industry (n = 12). The three most common organ sites for which assessment tools were available (breast, lung, colorectal) corresponded to the most common causes of cancer mortality among men and women in the United States [35]. This suggests that the assessment tools are responding to a public health need. However, three factors suggest that some of these websites might not be as useful to the public as they seem at first glance. First, many of the websites did not identify the statistical model used to calculate the risk estimate. Thus, visitors to these sites would have no way to verify that the risk assessment model had been scientifically vetted. The second factor that raises questions about the utility of some of these tools is the extensive use of undefined scientific terminology. Terms like “biopsy” and “radon progeny” may be confusing for individuals with limited literacy, thereby limiting their usefulness.

The third factor that raises concerns about these tools is the fact that they varied widely in their use of risk communication formats that facilitate comprehension of probabilistic information. For some risk communication formats, most websites followed experts’ recommendations (eg, 39 of 47 sites provided safety messages). For other formats, few websites followed recommendations (eg, 3 of 47 sites used positive and negative framing). It is unclear whether it is necessary to include all of the risk communication formats identified in Table 1 in a single communication effort. Indeed, it is possible that doing so would overwhelm people with too much information and bias their risk perceptions [38]. However, risk perceptions can also be biased if people receive too little information. For example, seven websites provided information seekers with a simple count of risk factors or a “risk score.” One such website then informed people, “You should do something to reduce your risk,” but it did not provide specific safety messages, such as “stop smoking.” Telling people to “do something” without providing specific recommendations does not facilitate comprehension [33] and might be counterproductive [32,37].

Two websites underscore the potential pitfalls of communicating risk using only numbers or only words. One of these websites provided two numerical risk estimates: one represented the risk if the information seeker continued the health-damaging behavior, and the other represented the risk if the individual stopped the behavior. However, the absolute risk was low in both cases (2% if the behavior continued and 1% if the behavior stopped), which might discourage people from changing their behavior [24,33,39]. The second website provided risk information as words only. It described cancer risk as “moderate,” “increased,” “high,” and “highest.” There was no “low risk” category. Thus, an 18-year-old with no risk factors for the type of cancer addressed on this website would be informed that he has a “moderate” risk of cancer. The combination of using only words to describe a risk and using the word “moderate” to describe the lowest-risk category might exaggerate risk perceptions among people at lowest risk [40].

It is also important to note that online risk assessment tools often have different objectives. For example, some risk assessment tools are designed to educate people about healthy lifestyles, whereas others are designed to persuade people to use their services, purchase their products, or engage in a particular health behavior. While it may be acceptable to attempt to persuade people to purchase necessary goods or services, or to engage in appropriate health behaviors, it is incumbent on developers of cancer risk assessment tools to consider the point at which persuasion may infringe upon an individual’s ability to make an informed decision. It is beyond the scope of this study to assess the underlying motivations of the various risk assessment tool developers, but website developers, clinicians, researchers, and public health officials should consider the ethical implications of the tools they design.

Despite these concerns, cancer risk assessment tools have the potential to play an important role in cancer prevention and control. However, in order to be acceptable and effective, they should use language that is appropriate for low-literacy populations, communicate risk estimates using formats that increase comprehension and reduce biased interpretations of risk, and provide information about the model used to calculate the estimate.

As mentioned previously, the communication formats chosen will depend upon the goals of the communication. For example, if a developer’s goal is to educate women about how the risk of breast cancer increases with age, the ideal website might use a line graph to portray how her risk changes over time [27]. It might describe in words how the risk changes and add the probability estimates at 5-year intervals. It might also include lines that depict the “average woman’s” risk over the same time period and the risk of heart disease over the same time period. The website would include accurate information about how often women should undergo mammography screening, how to obtain a mammogram, and how to maintain a healthy weight. A statement would inform visitors that the risk estimate contains some uncertainty, and it would identify the statistical model that was used to generate the risk estimate. Finally, before the website is released to the public, it would be tested for readability and comprehensibility among people with low literacy and to ensure that it elicited relatively unbiased risk perceptions. If it was found to be confusing, the website developer might consider removing some of the information, such as how the woman’s risk of breast cancer compared to her risk of heart disease. It might also test whether providing users with control over the amount of information displayed—such as being able to add or remove the 5-year probability estimates—results in better outcomes.

### Strengths, Limitations, and Future Directions

This content analysis fills a gap in existing research on Internet-based cancer risk assessment tools. This is the first study to describe how these tools communicate individualized risk estimates to the public and to examine the relationship between risk communication format and developer affiliation. Furthermore, this study replicated the finding that information needed to evaluate the quality of the risk estimate is often missing from these tools [5].

Our extensive search methodology increases the likelihood that most of the Internet-based cancer risk assessment tools that were available in mid-October 2007 were included in this analysis. However, since then, some tools might have added or removed features, additional websites might have been activated, and existing websites might have been deactivated. The small number of websites precluded formal statistical analysis of affiliation-based differences in communication formats. Furthermore, we only evaluated the presence or absence of specific risk formats. We did not evaluate whether the websites implemented the risk communication formats appropriately. To the best of our knowledge, empirical research has not identified the relative importance of each of the communication formats examined in this study. Consequently, we did not evaluate the overall quality of each website, nor can we say that using a greater number of risk communication formats results in better comprehension than using a lower number of formats.

 Future research should examine the demographic and cancer risk profiles of people who use Internet-based cancer risk assessment tools to identify how these tools influence cancer-related cognitions, emotions, and behaviors. This will be increasingly important as we enter the second generation of the Internet [41]. Web 2.0 is eroding the conventional boundaries between information providers and information seekers. The move from static personal websites to blogs, from online encyclopedias to Wikipedia, and from one-player video games to complex interactive virtual worlds exemplifies this transformation. The Centers for Disease Control and the American Cancer Society already have virtual outposts in the online world Second Life [42]. The interactive capabilities of Web 2.0 to help people understand cancer risk and cancer risk reduction strategies may be an important means to promote a healthy lifestyle.

### Implications

Internet-based cancer risk assessment tools have the potential to reach a wide audience and motivate people to engage in cancer preventive behaviors. However, because the tools do not always communicate risk in ways that facilitate comprehension, patients may misperceive or be confused about their cancer risk. This confusion may result in unintended negative consequences, such as failing to seek appropriate medical care. Researchers, organizations and clinicians who wish to provide risk assessment services to the public or their patients should refer to several excellent reviews [20,22,25] for specific recommendations on communicating risk in ways that facilitate comprehension, rather than foment confusion.

## Abbreviations

HINTS: Health Information National Trends Survey
